# Improving screening methods for psychosis in an adolescent help-seeking population using the Child Behavior Checklist (CBCL) and the Youth Self Report (YSR) versus the Prodromal Questionnaire -16 items version (PQ-16)

**DOI:** 10.1186/s13034-022-00459-w

**Published:** 2022-03-31

**Authors:** Yvonne de Jong, Albert E. Boon, Daniek Gouw, Mark van der Gaag, Cornelis L. Mulder

**Affiliations:** 1 Parnassia Psychiatric Institute, Rotterdam and The Hague, the Netherlands; 2 Department of Psychiatry, Epidemiological and Psychiatric Research Institute, Erasmus MC, Rotterdam, the Netherlands; 3 LUMC Curium - Child and Adolescent Psychiatry, Leiden University Medical Center, Leiden, The Netherlands; 4 Department of Clinical Psychology, Vrije Universiteit, Amsterdam, the Netherlands

**Keywords:** Early detection, Adolescence, PQ-16, CBCL, YSR, UHR, CHR-P, Psychosis

## Abstract

**Background:**

Screening methods for detecting Ultra High Risk status (UHR) or psychosis should be improved, especially in adolescent samples. We therefore tested whether the Child Behavior Checklist (CBCL) and the Youth Self Report (YSR) add value to the Prodromal Questionnaire-16 items version (PQ-16) for detecting UHR status or psychosis.

**Methods:**

We included help-seeking adolescents who had completed the PQ-16, YSR, CBCL, and a Comprehensive Assessment of an At Risk Mental States (CAARMS) interview, and used independent samples t-tests and binary logistic regression analyses to determine the scales contributing to the prediction of UHR status or of having reached the psychosis threshold (PT). Cutoff scores were determined using ROC analyses.

**Results:**

Our sample comprised 270 help-seeking adolescents (mean age 14.67; SD 1.56, range 12–17); 67.8% were girls and 66.3% were of Dutch origin. The Thought Problems syndrome scales of both the YSR and the CBCL best predicted UHR or PT, and had screening values comparable to the PQ-16. Other syndrome scales did not improve screening values. Although combining measures reduced the number of false negatives, it also increased the number of adolescents to be interviewed. The best choice was to combine the YSR Thought Problems scale and the PQ-16 as a first-step screener.

**Conclusions:**

Combining measures improves the detection of UHR or PT in help-seeking adolescents. The Thought Problems subscales of the YSR and CBCL can both be used as a first-step screener in the detection of UHR and/or psychosis.

*Trial registration* Permission was asked according to the rules of the Ethics Committee at Leiden. This study is registered as NL.44180.058.13

## Background

Increasing evidence shows that psychotic-like experiences (PLEs)—experiences such as perceptual anomalies, unusual beliefs and distorted thinking [1]—occur in all disorders and do not predict imminent transition to psychosis [2]. However, persistent PLEs predict the risk of psychosis [3]. Other pathways that lead to psychosis without preceding psychotic symptoms have also been reported; as well as depression, anxiety, bipolar disorder, and obsessive compulsive disorder [4, 5], they include thought disorder, somatic symptoms, attention problems and behavior disorder [6–8].

The concept of Ultra High Risk (UHR) status, also known as Clinical High Risk State for Psychosis (CHR-P [9]), has proved its clinical relevance in detecting psychosis risk in those seeking help for mental problems, being distressed and functionally impaired [10, 11]. Screening methods for UHR and psychosis nonetheless need to be improved, especially for adolescents, in whom most PLEs are mild and transient [12, 13], although attenuated PLEs in adolescence are predictive of psychosis [14]. It should also be noted that episodes of untreated psychosis are more common in adolescent-onset psychosis than in adult-onset psychosis [15], and that they are associated with higher comorbidity, functional impairment, and poorer illness outcome [16]. However adolescent-onset psychosis may have an equally good or even better outcome than adult-onset psychosis [15, 17], which would indicate that early detection of either UHR or Psychosis Threshold (PT) is particularly important in adolescence.

Another reason it is important to screen for UHR and psychosis in adolescents is that psychotic disorders are difficult to detect during adolescence, due not only to their phenomenological overlap with affective symptomatology [18–21], but also because their psychopathology in general is less developed and therefore less distinguishable [22]. Family members and healthcare professionals may be more inclined to attribute problematic behavioral and emotional problems to puberty and may overlook the presence of psychotic symptoms [15]. In view of the various pathways to psychosis, we hypothesized that screening methods for UHR or psychosis in adolescence might be improved by combining the 16-item version of the Prodromal Questionnaire (PQ-16; [23] with the screening tools for present comorbid psychopathology already used in clinical practice, such as the commonly used Achenbach System of Empirically Based Assessment (ASEBA [24, 25]).

The PQ-16 is used mainly in a two-step screening procedure with the Comprehensive Assessment of At Risk Mental States CAARMS; [26], an interview for determining a CAARMS classification of UHR or PT [23, 27, 28]. Although the PQ-16 has acceptable psychometrics in youth seeking help, its specificity rates need to be improved [29–31].

The multi-informant ASEBA questionnaires [24] are used in many Child and Adolescent Mental Health Services (CAMHS) as instruments for completely and accurately assessing psychopathology in children and adolescents [32]. These instruments include the Youth Self Report (YSR), which is filled out by adolescents aged 11–18; and the Child Behavior Checklist 6–18 years old (CBCL), which is filled out by parents. The reliability and validity of the ASEBA questionnaires are well established [25, 33]. In a sample of help-seeking 6 to 18-year-olds [34], the CBCL Thought Problems scale was found to be diagnostically efficient in screening for psychotic disorders, as confirmed by the Schedule for Affective Disorders and Schizophrenia Psychosis items (K-SADS). In a general student sample of 12 to 18-year-olds, the CBCL Thought Problems and Withdrawn/depressed scales were also found to be the most discriminating between UHR and other groups [7].

To our knowledge, the ability of the syndrome scales of the CBCL and the YSR to detect an UHR status or psychosis have never previously been tested in combination with the PQ-16 and the CAARMS. For this reason, in a two-step screening procedure with the CAARMS, the values of the CBCL were compared with those of the YSR, both singly and in combination with the PQ-16, for predicting UHR or psychosis.

## Methods

### Aim

To improve the early detection of psychosis in a help-seeking adolescent population by comparing the predictive power for UHR or psychosis of the CBCL and the YSR, both alone and in combination with the PQ-16.

### Setting and design

All participants were help-seeking adolescents who had been referred for assessment and treatment at Youz, an outpatient CAMHS, between 20 February 2014 and 16 January 2018. Adolescents aged 12–18 were included if they had been given a psychiatric DSM-IV or a DSM-5 classification after a face-to-face intake procedure performed by a psychiatrist or clinical psychologist working with another trained healthcare professional. Adolescents without a DSM classification were referred to the non-psychiatric youth services. As part of a standard monitoring system, newly referred adolescents were asked to complete the YSR and the PQ-16, and their parents were asked to complete the CBCL. To prevent stigma during the screening stage, we used the term “screening for unusual experiences” rather than “screening for psychotic symptoms.” More details of these selection procedures have been reported elsewhere [31].

**Fig. 1:**
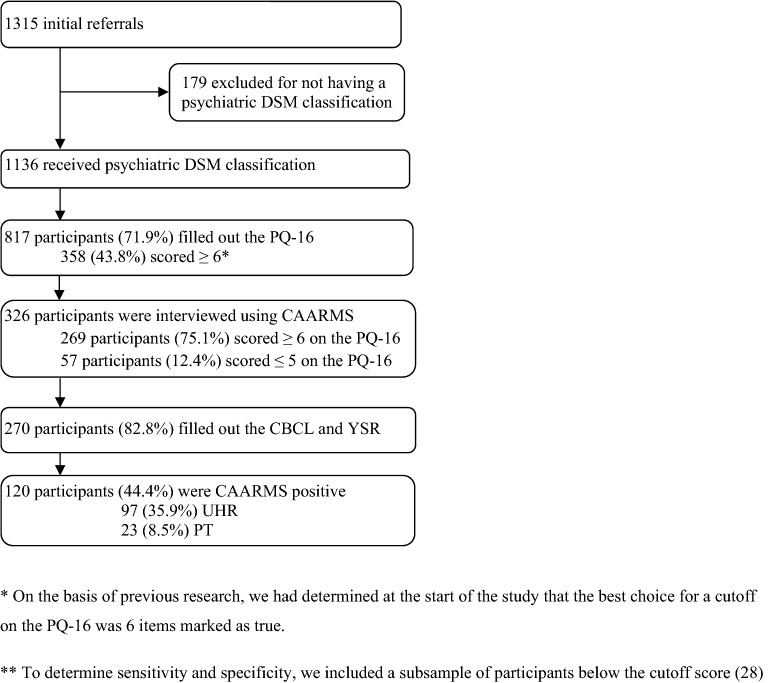
Participant flowchart

On the basis of earlier cutoff definitions [23, 28], adolescents who scored ≥ 6 on the PQ-16 were interviewed using the CAARMS. To be able to determine sensitivity and specificity for detecting UHR and PT, we interviewed a subsample who had scored ≤ 5 on the PQ-16. To be included in our research sample, participants had to have completed a PQ-16, YSR, CBCL and a CAARMS interview. Figure 1 shows a flowchart of selected participants.

### Measurements

#### PQ-16

The Prodromal Questionnaire-16 items version (authorized Dutch translation [23] is a self-report screening questionnaire that assesses the presence of positive and negative symptom items on a 2-point scale (true/false). The total score on the PQ-16 is calculated by adding up all agreed items [31]. Depending on the setting in which the PQ-16 is administered, cutoff scores differ so as to minimize false positives [28]. However, on the basis of previous research, we decided at the start of our study that the best choice for a cutoff on the PQ-16 was 6 items marked as true. We later showed that a cutoff of ≥ 7 items was more appropriate for help-seeking adolescents aged 12–17 [31].

#### CAARMS

The Comprehensive Assessment of At Risk Mental States (CAARMS) (Yung, Yuen et al. 2005 [26]) is a semi-structured clinical interview that is frequently used to identify patients at risk for a first psychotic episode or to identify patients already experiencing a psychotic episode. Upon completion of this assessment by experts and trained clinicians, referred subjects are assigned a status of not at risk, at ultra-high risk (UHR), or as having reached the psychosis threshold (PT). To reach consensus, trained CAARMS interviewers frequently met to discuss and assess cases. All interviewers were blind to the PQ-16 score. Participation was voluntary.

#### ASEBA (CBCL, YSR)

The Achenbach System of Empirically Based Assessment (ASEBA; [24] is a multi-informant assessment system. It comprises 113 corresponding items that cover a broad range of emotional and behavioral problems experienced in the past 6 months, which are displayed in eight syndrome scales [25]. The items are rated from 0 (not true) to 2 (very true or often true). Adolescents fill out the YSR and their parents fill out the CBCL. Because the CBCL is usually filled out by the mother, our first choice was to analyze a CBCL filled out by the mother. But in 33 cases, it had been filled out by the father. The CBCL and YSR scale scores offer a *T*-score based on age and sex norms.

### Statistical analysis

As the PQ-16 contains items without a scale for the seriousness of the symptoms, it does not discriminate between severe and persistent or mild and transient symptoms [23]. In our sample, effect sizes of Cohen’s d were between d = − 0.5 and d = 0.5; 95%-CI. For that reason, the UHR and the PT groups were combined in a single group, the CAARMS- positive group.

Independent samples t-tests were performed on the ASEBA syndrome scale total scores between the groups of participants with and without an UHR or PT. To determine which independent variables significantly predicted the CAARMS outcome, [1] the ASEBA syndrome scales with a medium and large effect size (Cohen’s d > 0.5, [35]) on the t-test were entered with the total scores of the PQ-16 as independent variables in a logistic regression analysis (forward method); and [2] the CAARMS-negative outcome (no classification) or positive outcome (UHR or PT) were entered as the dependent variable. To control the outcome, a logistic regression analysis (backwards method) was performed. To compare the areas under the curve (AUC) of the significant predictors with the CAARMS outcome as state variable, we performed a Receiver Operating Characteristic (ROC) analysis using the syndrome scale scores of the CBCL and YSR with a high effect size and the total scores of the PQ-16. AUC scores above 0.70 are considered clinically useful [36]. We aimed to include as many adolescents as possible with a sensitivity not lower than 0.80 and an acceptable level of false positives. Since a false positive is not followed by an invasive procedure, but by an interview, we chose as moderate a specificity as permissible, eliminating false positives of those who scored ≥ the chosen cutoff score on the PQ-16 [30, 37]. Crosstabs between the CAARMS outcome and the determined cutoffs of the PQ-16, YSR and CBCL were used to determine the proportions of true and false positives and negatives.

## Results

### Sample

Table 1 shows sample characteristics. The PQ-total scores in the eligible sample (*n* = 270) did not differ from those in the sample of adolescents who had been interviewed with the CAARMS but had not completed the CBCL and YSR (n = 56) (*t(324)* = 1.45, *p* = 0.15). Neither did these samples differ significantly in the CAARMS classification: χ^2^ (2, *n* = 326) = 3.80, *p* = 0.15.

**Table 1:** Sample characteristics: age, gender, ethnicity, level of education, location of referral, and main DSM-IV or V classification (N = 270)

Characteristic	Mean	SD
Age total	14.67	1.56
Characteristic	N	%
Gender		
Male	87	32.2
Female	183	67.8
Ethnicity		
Dutch	179	66.3
Immigrant first generation	16	5.9
Immigrant second generation	70	25.9
Unknown	5	1.9
Level of education		
Elementary school	12	4.4
Special school	2	0.7
Secondary education	225	83.3
Vocational education	30	11.1
No education	1	0.4
DSM classification*		
ADHD	45	16.7
Autism	25	9.3
Mood disorder	90	33.3
Anxiety and compulsivity	39	14.4
Trauma and dissociation	12	4.4
Psychotic disorder	5	1.9
Eating disorder	5	1.9
Behavior and impulse	14	5.2
Substance abuse	4	1.5
Mental disorder nos**	77	28.5
Personality disorder	2	0.7

^*^DSM classification total prevalence including comorbidities

^**^Not otherwise specified

Neither was there a difference between the mean age of the total initial sample (*N* = 1136) and that of the eligible sample (*n* = 270) (*t(1134)* = -0.51, *p* = 0.61). However, the difference in gender was significant, as the percentage of girls in the eligible sample was significantly higher: χ^2^ *(*1, N = 1136) = 16.92, *p* < 0.001, Cohens’*d* = 0.25. Girls were also more likely to receive a CAARMS diagnosis χ^2^ (1, *n* = 270) = 6.42, p = 0.01. In addition, adolescents aged 14 years old (88% girls) and 15 years old (68% girls) were more likely to receive a CAARMS diagnosis: χ^2^ (5, n = 270) = 22.68, *p* < 0.001.

### CBCL and CAARMS outcome

All syndrome scales total scores of the CBCL were compared for the CAARMS-negative and positive outcome groups (see Table 2). The Thought Problems scale (hereafter CBCL-T) was associated with CAARMS outcomes with a large effect-size. Somatic Problems also showed a statistically significant medium effect (See Table 2).

**Table 2:** Comparison of PQ-16 and syndrome scales of CBCL and YSR with negative or positive CAARMS outcomes (no classification versus UHR or PT)

CBCL	CAARMS negative (N = 150)		CAARMS positive (N = 120)				
	*M*	*SD*	*M*	*SD*	*t (df* = *1)*	*p*	*d*
Anxious/depressed	66.4	10.7	70.0	11.1	− 2.71	.007	0.33
Withdrawn/depressed	69.3	11.0	73.3	10.4	−3.03	.003	0.37
Somatic Problems	64.3	9.3	70.2	9.9	−5.08	< .001	0.62*
Social Problems	62.9	9.2	64.0	8.6	−0.93	.356	0.11
Thought Problems	65.5	8.4	71.2	6.1	−6.33	< .001	0.75*
Attention Problems	65.5	9.7	64.6	9.7	0.73	.463	0.09
Rule − breaking Behavior	59.6	7.8	59.1	8.0	0.50	.617	0.06
Aggression	61.7	10.2	60.4	9.4	1.12	.263	0.14
YSR	CAARMS negative (N = 150)		CAARMS positive (N = 120)				
	*M*	*SD*	*M*	*SD*	*t (df* = *1)*	*p*	*d*
Anxious/depressed	66.5	12.1	73.8	12.0	−4.95	< .001	0.61*
Withdrawn/depressed	66.8	12.1	73.0	11.5	−4.31	< .001	0.53*
Somatic Problems	62.4	8.6	67.6	9.8	−4.63	< .001	0.57*
Social Problems	62.9	8.3	66.4	7.9	−3.48	< .001	0.43
Thought Problems	61.6	8.1	69.2	8.8	−7.41	< .001	0.91*
Attention Problems	66.0	11.1	68.3	10.4	−1.74	.082	0.21
Rule − breaking Behavior	57.5	6.6	58.9	6.5	−1.79	.075	0.22
Aggression	58.1	8.2	58.0	7.2	0.15	.880	0.02
PQ − 16	CAARMS negative (N = 150)		CAARMS positive (N = 120)				
	*M*	*SD*	*M*	*SD*	*t (df* = *1)*	P	d
Total score	7.26	3.2	9.83	3.2	− 6.56	< .001	0.80*

^*^These scales were entered as independent variables in the logistic regression analysis

### YSR and CAARMS outcome

The Thought Problems scale (hereafter YSR-T) was associated with the CAARMS outcome and had a large effect size. Anxious/depressed, Social Problems and Withdrawn/depressed had medium effect-sizes (see Table 2).

### PQ-16 and CAARMS outcome

The PQ-16 total scores were associated with the CAARMS outcome and had a large effect-size (See Table 2).

### Determination of predictive values of CBCL, YSR syndrome scales, and PQ-16 total score for determining CAARMS classification

The scales with a medium or large effect size (see Table 2) were entered as independent variables in a binary logistic regression analysis (forward method). After three steps, a satisfactory solution (Nagelkerke *R*^2^ = 0.342) was found, the most predictive of a CAARMS classification being PQ-16 total score, *OR* = 1.19, sig < 0.001; CBCL-T, *OR* = 1.09, sig < 0.001; and YSR-T, *OR* = 1.06, sig = 0.002. A binary logistic regression analysis, backwards method, had a comparable outcome, in which the same scales were significant (*OR* = 1.07–1.18, *p* = 0.001–0.003). There was no age effect found for the different measures. Box 1 shows the items of the CBCL Thought Problems scale versus the YSR Thought Problems scale.

Box 1. The items of CBCL thought problems versus the items of YSR thought problems

**Table:** 

CBCL item number	CBCL item content	YSR Item number	YSR item content
9	Can’t get his/her mind off certain thoughts; obsessions	9	I can’t get my mind off certain thoughts
18	Deliberately harms self or attempts suicide	18	I deliberately try to hurt or kill myself
40	Hears sound or voices that aren’t there	40	I hear sounds or voices that other people think aren’t there
46	Nervous movements or twitching	46	Parts of my body twitch or make nervous movements
58	Picks nose, skin, or other parts of body	58	I pick my skin or other parts of my body
59	Plays with own sex parts in public	–	–
60	Plays with own sex parts too much	–	–
66	Repeats certain acts over and over; compulsions	66	I repeat certain acts over and over
70	Sees things that aren’t there	70	I see things that other people think aren’t there
76	Sleeps less than most kids	76	I sleep less than most kids
83	Stores up too many things he/she doesn’t need	83	I store up too many things I don’t need
84	Strange behavior	84	I do things other people think are strange
85	Strange ideas	85	I have thoughts that other people would think are strange
92	Talks or walks in sleep	–	–
100	Trouble sleeping	100	I have trouble sleeping

### Determination of cutoff scores

**Fig. 2:**
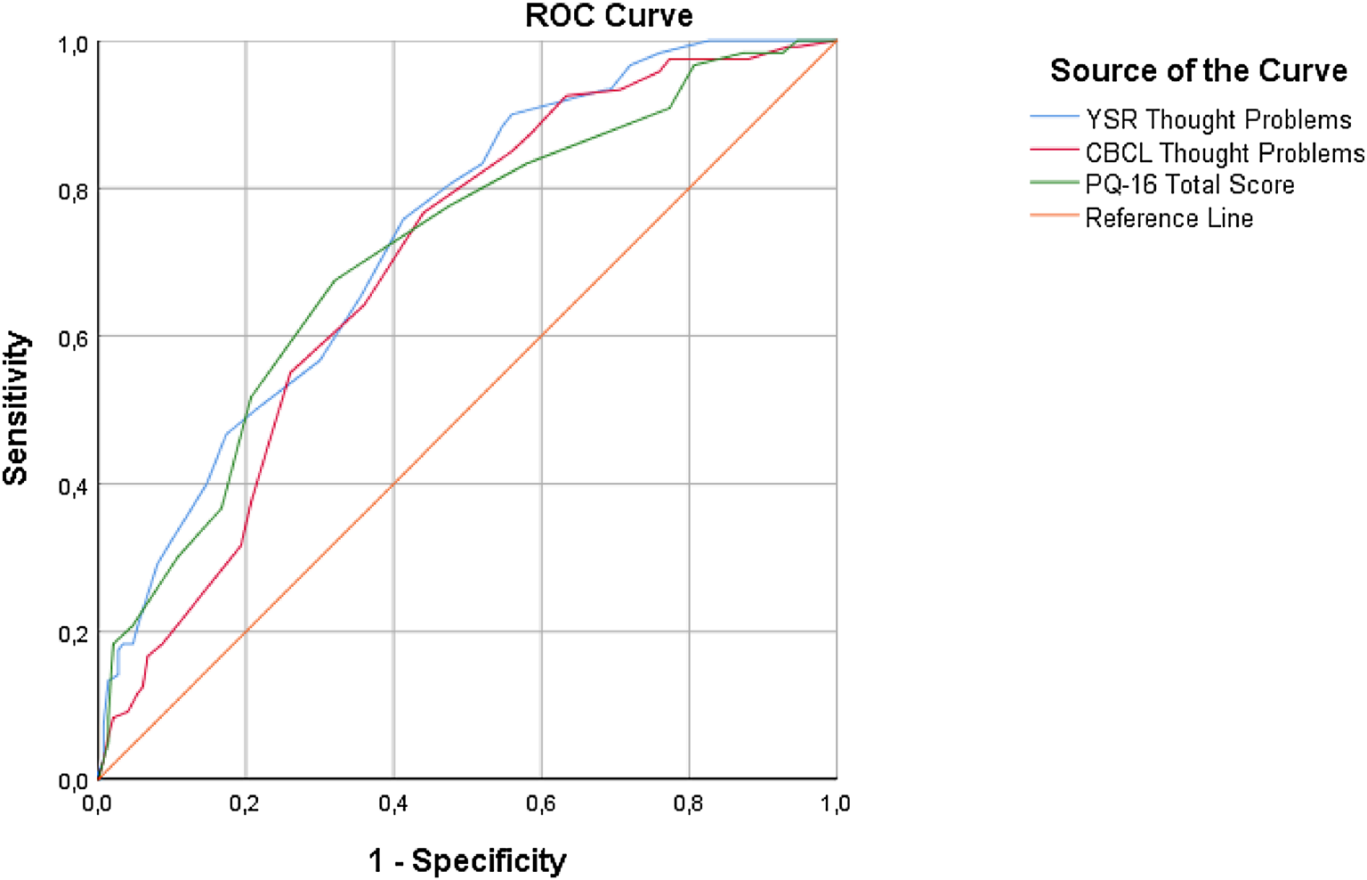
ROC curves of YSR Thought Problems, CBCL Thought Problems, and PQ-16 total score

Figure 2 shows the ROC curves indicating the predictive ability of the total scale scores of YSR-T, CBCL-T and PQ-16, versus the CAARMS outcome. All measures discriminated well between CAARMS-negative and CAARMS-positive outcome. The AUC for YSR-T = 0.74, (95% CI 0.68–0.79, p < 0.001). The AUC for CBCL-T = 0.70, (95% CI 0.64–0.76, p < 0.001). The AUC for PQ-16 total score = 0.71 (95% CI 0.65–0.78, p < 0.001). When determining a cutoff score, we chose the first cutoff that reached good sensitivity (≥ 0.80), and thus included as many adolescents as possible, while generating an acceptable level of false positives. As stated above, we considered a moderate specificity to be permissible, since a false positive is not followed by an invasive procedure.

For the YSR-T, we chose the cutoff = 63; for the CBCL-T we chose = 67; and for the PQ-16 total score we chose = 7 (see Table 3). Our results indicated that PQ-16, YSR-T and CBCL-T had comparable screening values, with the difference that a higher percentage of children and adolescents should be interviewed when the CBCL-T was used as a screener.

**Table 3:** Screening properties of the PQ-16, YSR Thought Problems, CBCL Thought Problems and positive CAARMS outcome (UHR/psychotic threshold) vs. negative CAARMS outcome (no classification), with two cutoff values above and below the chosen cutoff

Questionnaire / cutoff	N reaching the cutoff n = 270	N and % reaching the cutoff in total sample n = 1136	Sensitivity	Specificity	PPV	NPV	True pos	True neg	False pos	False neg
YSR Thought Problems										
61	188	393 34.6	0.88	0.45	56.4	82.9	106	68	82	14
62	178	362 31.9	0.83	0.48	56.2	78.3	100	72	78	20
63	169	332 29.2	0.81	0.52	57.4	77.2	97	78	72	23
64	153	288 25.4	0.76	0.59	59.5	75.2	91	88	62	29
65	131	227 20.0	0.65	0.65	59.5	69.8	78	97	53	42
CBCL Thought Problems										
64	206	614 54.0	0.93	0.37	53.9	85.9	111	55	95	9
65	193	563 49.6	0.88	0.41	54.4	80.5	105	62	88	15
67	186	524 46.1	0.85	0.44	54.8	78.6	102	66	84	18
68	158	427 37.6	0.77	0.56	58.2	75.0	92	84	66	28
70	131	345 30.4	0.64	0.64	58.8	69.1	77	96	54	43
PQ-total score										
5	237	437 38.5	0.97	0.19	48.9	87.9	116	29	121	4
6	225	357 31.4	0.91	0.23	48.4	75.6	109	34	116	11
7	187	287 25.3	0.83	0.42	53.5	75.9	100	63	87	20
8	164	239 21.0	0.78	0.53	56.7	74.5	93	79	71	27
9	129	185 16.3	0.68	0.68	62.8	72.3	81	102	48	39

*PPV*  *Positive Predictive Value, NPV*  *Negative Predictive Value, pos* *positives, neg* *negatives*

### Combining measures to improve prediction of a CAARMS outcome

**Fig. 3:**
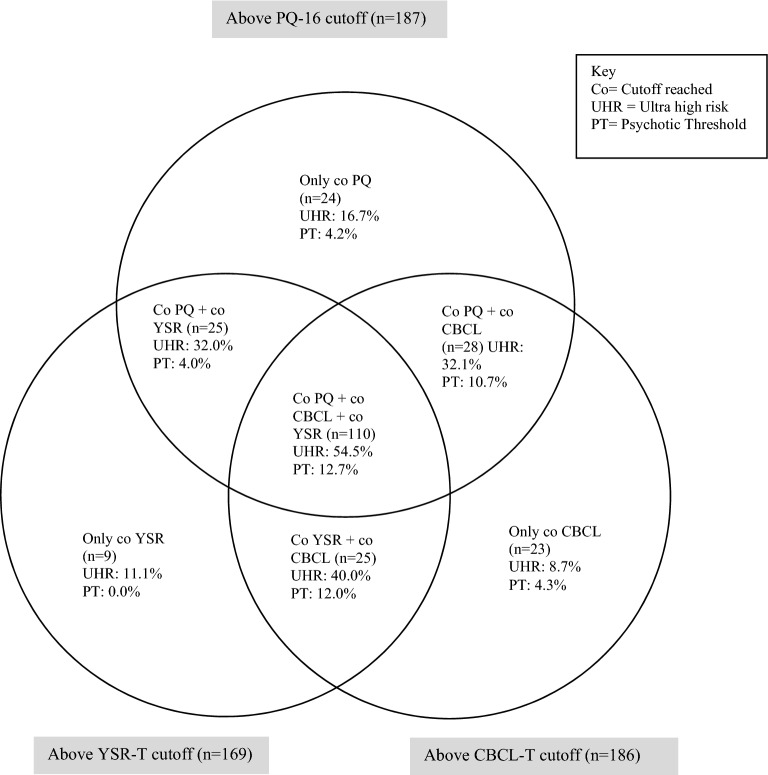
VENN diagram of the groups that reached the cutoff score on one or more measures

Figure 3 shows a VENN diagram of the various groups that reached the cutoff scores chosen for the measures shown in Table 3. Table 4, section A, shows the screening values for these different groups. When distinguishing between participants who had reached the cutoff scores on all measures and those who had not, the Positive Predicted Value (PPV) for positive CAARMS was 67.3, but relatively more false negatives (n = 46) were produced than by the PQ-16 alone (n = 20). Only 3 false negatives remained after all participants who reached the cutoff score on one or more of the measures for an interview had been distinguished from those who did not reach it. In this selection procedure, however, the PPV was 48.0, which was slightly lower than the PPV of the PQ-16 only (53.5). In addition, false positives were higher when all measures were used than when only the PQ-16 was used (127 vs. 87); and more adolescents had to be interviewed using the three measures (59.3%) than using the PQ-16 alone (25.3%).

**Table 4:** Screening values of a CAARMS classification yielded by Section A the various combinations of the measures and section B a sequential two step method

Cutoff scores reached on the measure(s)*	*N reaching the cutoff n* = *270*	*N and % reaching the cutoff total sample* *n* = *1136*	*Sens*	*Spec*	*PPV*	*NPV*	*True positives*	*True negatives*	*False positives*	*False negatives*
Section A: various combinations of measures										
Did not reach any cutoff	26	191 16.8	–	–	–	–	–	–	–	–
** ↑**PQ ↓CT ↓YT**	24	34 3.0	0.04	0.87	20.8	53.3	5	131	19	115
↓PQ ↓CT ↑YT	9	33 2.9	0.01	0.95	11.1	54.4	1	142	8	119
↓PQ ↑CT ↓YT	23	146 12.9	0.03	0.87	13.0	52.6	3	130	20	117
↑PQ ↓CT ↑YT	25	31 2.7	0.08	0.89	36.0	54.7	9	134	16	111
↑PQ ↑CT ↓YT	28	37 3.3	0.10	0.89	42.9	55.4	12	134	16	108
↓PQ ↑YT ↑CT	25	82 7.2	0.11	0.92	52.0	56.3	13	138	12	107
↑PQ ↑YT ↑CT	110	131 11.5	0.62	0.76	67.3	71.3	74	114	36	46
PQ and/or YT	221	449 39.5	0.95	0.29	51.6	87.8	114	43	107	6
PQ and/or CT	235	624 54.9	0.97	0.21	49.4	88.6	116	31	119	4
YT and/or CT	220	613 54.0	0.93	0.28	50.9	84.0	112	42	108	8
PQ and/or YT and/or CT	244	674 59.3	0.98	0.15	48.0	88.5	117	23	127	3
Section B: a sequential two step method										
(1) ↑ CT (2) ↑ PQ	186 138	524 46.1 187 16.5	0.72	0.65	62.3	74.2	86	98	52	34
(1) ↑ YT (2) ↑ PQ	169 135	332 29.2 170 15.0	0.69	0.65	61.5	72.6	83	98	52	37
(1) ↑ CT and/or ↑ YT (2) ↑ PQ	220 163	613 54.0 226 19.9	0.79	0.55	58.3	76.6	95	82	68	25

^*^Cutoff scores reached on the PQ-16 total score (PQ), CBCL Thought Problems total scale score (CT) and YSR Though Problems total scale score (YT)

^**^↑ means ≥ cutoff ↓ means < cutoff

^***^(1) and (2) represent a sequential method; in the first step a reached cutoff at CT or YT was selected and in the second step a reached cutoff at the PQ was selected

A selection procedure using the PQ-16 in combination with the YSR-T (PQ and/or YT; see Table 4) or the CBCL-T (PQ and/or CT; see Table 4) improved the screening procedure, since combining these measures produced more true-positive cases and fewer false-negative cases than when the PQ-16 was used alone. The number of adolescents who had to be interviewed using the CAARMS increased more when the CBCL-T was combined with the PQ-16 (54.9%) than when the PQ-16 was used alone (25.3%) and when the YSR-T was added to the PQ-16 (39.5%). However, although a CAARMS classification was given to 13.0% of the adolescents who had been selected solely because their parents had reached the cutoff on the CBCL-T (Fig. 3), these adolescents were not detected by the PQ-16 and/or YSR-T.

A selection procedure using a sequential method also seems to improve the screening procedure (see Table 4 section B). In this procedure first adolescents that had reached a cutoff at the CBCL-T or YSR-T were selected and in this sample at the second step adolescents that had reached the cutoff at the PQ-16 were selected. If one or both of the cutoffs of the CBCL-T or YSR-T were reached and the reached cutoff of the PQ-16 was added, the screening values were comparable with using the PQ-16 alone. Although this procedure produced 5 more false negatives, it also produced 19 less false positives and a lower percentage of the total group that had to be interviewed in the next step (19.9% vs 25.3%). However, this method had less true positives and more false negatives than a selection procedure using the PQ-16 in combination with the YSR-T.

## Discussion

We compared the ASEBA child and parent syndrome scale scores with the PQ-16 total scores and cutoff scores, in combination or in a sequential procedure, for their ability to predict the CAARMS classification (UHR and PT combined). UHR/PT status was best predicted in a sample of 270 help-seeking adolescents aged 12–17 by the Thought Problems scale of both the YSR and the CBCL. Although both had screening values comparable to those of the PQ-16 for predicting the outcome of the CAARMS, a considerably higher percentage of adolescents needed to be interviewed using the CAARMS when the CBCL was added to the PQ-16 as a screener. However, if the CBCL or the YSR are taken first and the PQ-16 is added in the second step, the screening values ​​are comparable to the PQ-16 alone and fewer adolescents need to be interviewed.

It is unclear why parents reported more thought problems than adolescents. Agreement between the Thought Problems scales of YSR and CBCL is generally low [24, 25, 33]. It is possible that parents see thought problems as symptoms of other problems and overlook the PLEs [38]. Adolescents, for their part, seem to find it easier to disclose PLEs when filling out a screener [39].

When used by itself, each of these measures shows similar low values for predicting UHR/PT status. They have added value when combined as a first-step screener with the PQ-16 for detecting UHR and/or psychosis preceding the CAARMS.

The cutoff scores and screening values found in this study show which measure or measures could be chosen as a first-step screener for UHR or psychosis. To our knowledge, this is the first time that cutoff scores for the YSR and CBCL have been determined in relation to the CAARMS. The cutoff score in this sample of 7 or more agreed items on the PQ-16 is in agreement with the cutoff score we found previously [31].

We also presented predictive values of scores that can be used when the measures are present in the regular assessment procedures of a CAMHS. As a standard first-step screener in adolescents, it seems better to combine the YSR Thought Problem syndrome scale with the PQ-16 than to use the PQ-16 alone, as the combination produces more true-positive and fewer false-negative cases, and the smallest rise in the number of adolescents who have to be interviewed. Combining the YSR-T and the PQ-16 showed better screening values than a sequential method where the PQ-16 is only administered after having reached the cutoff on the YSR-T or CBCL-T. Adolescents who reach the cutoff score on all three measures (PQ-16 ≥ 7, CBCL-T ≥ 67, YSR-T ≥ 63, 11.5% of the total sample) should definitely proceed to the second-step interview with the CAARMS, since two thirds of them would be diagnosed with UHR or as heaving reached the psychotic threshold.

Our results showed that the combination of the PQ-16 and the Thought Problem scales had the highest number of true-positive cases and a very low number of true-negative cases. This is the quality preferred for a first-step screener: high sensitivity and an acceptable number of false-positives to be removed in the second classification step. These scales all contain the PLE Hearing and Seeing Things items, which were found to be the most predictive of psychosis [34, 40]. It remains unclear, however, which items in the Thought Problem scales are the most responsible for their predictive power.

The screening values of the CBCL in our study were comparable with those found by Salcedo and colleagues when using the CBCL-T only to detect clinically significant psychotic symptoms assessed with the K-SADS in a youth help-seeking sample [34]. In their study they found an AUC of 0.65 (versus 70 in ours), and a cutoff on the CBCL-T of 68.5 (versus 67 in ours). The screening values found in our study were also in line with those found by Thompson and colleagues [41] in a sample of 12–22 years old. They used the Behavior Assessment System for Children, second edition (BASC-2) to predict high risk status or psychotic disorder assessed by the Structured Interview for Psychosis-Risk Syndromes (SIPS). They found that the Atipicality scale of the BACS was most predictive. This scale is similar to the Thought Problems scale of both CBCL and YSR, because of the items related to seeing and hearing things. They also found reported PLEs by adolescents to be more predictive than those reported by parents.

The comparably low predictive values of the PQ-16, YSR and CBCL separately may have been due to the cross-sectional nature of our study. A recent prospective meta-analysis [42] showed that 25% of individuals at clinically high risk of psychosis developed psychosis within 3 years. The transition risk continued to increase over time and remained low for the first year. Another issue is that screening values also depend on gender and the level of enrichment of the research sample. In our sample we detected significantly more girls (67.8%). Girls have been known to experience more PLEs [43]. Populations enriched for psychosis have better screening values [28]. Our sample was taken from a help-seeking population who had not been referred specifically for assessment of a psychotic disorder.

Unlike our study, Simeonova et al. [7] found in a general population sample that the level of withdrawn/depressed symptoms reported by the parents on the CBCL had the most discriminating power between UHR or not UHR. In our study Anxious/depressed symptoms reported by adolescents added to the prediction of UHR/ PT, but not as much as the thought problems reported by the parents and adolescents in our help-seeking sample. It is possible that, due partly to higher distress and lower general functioning, help-seeking adolescents experience more thought problems, and therefore seek help. Both our results and Simeonova’s results might be a subject for future research and may be a signal for clinicians to ask for PLEs when parents or adolescents report depressed symptoms.

### Implications for clinical practice

While our results show that combining the PQ-16 with the CBCL and YSR adds value to the early detection of UHR or psychosis, more time and money will be required to extend screening by including these measures. While the PQ-16 is in the public domain and available free of charge, the CBCL and YSR must both be purchased. However, both are often used in CAMHS, and provide a complete and accurate assessment of psychopathology in children and adolescents [32]. If both CBCL and YSR are present and administered first, the PQ-16 can be added to lower the number of adolescents who have to be interviewed. Any additional costs incurred by purchasing measures and training professionals to conduct the CAARMS are compensated by the long-term healthcare cost savings expected from early intervention [44, 45]. Additional costs in the detection phase must also be seen in the context of preventing psychosis, a serious condition with high comorbidity and morbidity that is often overlooked in the early stages [15, 18, 46]. As adolescent onset is associated more usually with a delay in treatment and therefore worse outcome, a more structured approach should be taken to screening for psychosis. The ASEBA assessments also contain the Adult Self-Report (ASR) for 18 to 59-year-olds, whose screening potential for UHR/PT is not yet known.

When healthcare professionals start screening in daily practice, they should be aware that the term UHR is used as a scientific term signifying a risk profile. In practice, however, psychotic experiences can be very normal and should therefore be approached as such. And because the term “psychotic experience” can be used in combination with effective psychoeducation only if the symptoms are disabling or cause a person to suffer, a more appropriate term for the phenomenon is “unusual experiences”.

If the YSR is available in daily practice, the YSR Thought Problem scale can be used before the CAARMS as a first-step screener with a cutoff at ≥ 67, sensitivity of 0.85, and specificity of 0.44. If YSR and PQ-16 are available, they can be combined as a first-step screener, and thus show more true positives than is possible with the PQ-16 alone. If both YSR and CBCL are available and if the cutoff on one of these measures has been reached, the PQ-16 can be added, with a cutoff of ≥ 7, a sensitivity of 0.79, and specificity of 0.55. If the YSR is not available, the PQ-16 can be used alone with a cutoff at ≥ 7, sensitivity of 0.83, and specificity of 0.42.

### Strengths and limitations

The strength of this study is that, to our knowledge, we were the first to use the YSR and CBCL to assess improvements in screening methods by comparing them individually, in combination with the PQ-16, and in relation to the CAARMS in a large sample of adolescents aged 12–17 who had been referred to a CAMHS without pre-selection or a focus on specific psychiatric disorders.

A first limitation is that we had no access to the item scores of the ASEBA questionnaires, and were therefore unable to measure the added value of the individual items.

A second limitation is that we did not assess the participants’ IQs or reading levels. Some items in the PQ-16 may be difficult for adolescents to interpret; if so, this may have produced more false positives vis-à-vis the CAARMS classification. Additionally, as some questions in the CAARMS are difficult for adolescents to understand, interviewers had to simplify questions for young adolescents. Although the possible influence of this is unclear, all interviewers were trained child-and-adolescent professionals, who sought consensus by meeting frequently to discuss and assess cases.

Third, it is not known whether completion of the PQ-16 or YSR is affected by parents’ perceptions, as these measures were completed without the supervision of a healthcare professional.

Fourth, our inclusion of adolescents on the basis of a DSM classification was not supported by a validated structured clinical interview. Instead, it was based by a clinician [1] on his or her evaluation of the signs and symptoms indicated by adolescents and their parents, and [2] on his or her evaluation of the adolescents’ and their parents’ answers to the ASEBA questionnaires.

A fifth limitation is that it is not known how many participants in our study who had been classified as UHR eventually converted to psychosis.

Finally, as our study focused on adolescents referred to a CAMHS, our results cannot be generalized to the general population.

## Conclusions

The Thought Problem scales of the YSR and CBCL are comparable to the PQ-16 as first-step screeners for determining whether a second-step CAARMS should be administered. Combining these measures produces the best prediction of a CAARMS classification of UHR or of reaching the psychotic threshold. It is also useful for a complete assessment of psychopathology in children and adolescents. Combining the YSR Thought Problem scale and the PQ-16 as a first-step screener seems to be the best choice, in view not only of the number of adolescents who have to be interviewed using the CAARMS, but also of the number of true-positive CAARMS diagnoses it produces, and the fact that it produces the lowest number of false negatives.

## Data Availability

The dataset used during the current study is available from the corresponding author on reasonable request.

## Abbreviations

ASEBA: Achenbach System of Empirically Based Assessment
AUC: Area Under The Curve
CAARMS: Comprehensive Assessment of At Risk Mental States
CAMHS: Child and Adolescent Mental Health Services
CBCL: Child Behavior Checklist
CBCL-T: Child Behavior Checklist-Thought Problems syndrome scale
CHR-P: Clinical High Risk for Psychosis
PLE: Psychotic Like Experiences
PT: Psychotic Threshold
PQ-16: Prodromal Questionnaire 16 items version
ROC: Receiver Operating Characteristics
UHR: Ultra High Risk
YSR: Youth Self Report
YSR-T: Youth Self Report -Thought Problem syndrome scale
